# Trichoscopic stages of dissecting cellulitis: a potential complementary tool to clinical assessment^☆^^☆☆^

**DOI:** 10.1016/j.abd.2019.10.008

**Published:** 2020-05-11

**Authors:** Daniel Fernandes Melo, Luciana Rodino Lemes, Rodrigo Pirmez, Bruna Duque-Estrada

**Affiliations:** a Center for Trichology Studies, Hospital Pedro Ernesto, Universidade do Estado do Rio de Janeiro, Rio de Janeiro, RJ, Brazil; b Outpatient Clinic of Capillary Disorders, Instituto de Dermatologia Professor Rubem David Azulay, Santa Casa de Misericórdia do Rio de Janeiro, Rio de Janeiro, RJ, Brazil

**Keywords:** Alopecia, Dermoscopy, Folliculitis

## Abstract

Dissecting cellulitis is a chronic, progressive, and relapsing inflammatory disease that predominantly affects the vertex and occiput of young Afro-descendent men. It starts with papules and pustules that evolve to nodules, abscesses, and cicatricial alopecia. This article illustrates the evolutive trichoscopy of dissecting cellulitis, from its early phase, through the abscess phase, to the fibrotic cicatricial phase. Trichoscopy complements clinical-pathological classification, representing a complementary tool useful in early diagnosis and monitoring of the patient during treatment.

Dissecting cellulitis (DC) is a rare, chronic, progressive, and relapsing inflammatory disease, with a predominance of histopathological neutrophilic infiltrate. It is more frequent in young Afro-descendent men in the vertex and occipital area,^1^ with papules and pustules that can develop into nodules and interconnecting abscesses, or even cicatricial alopecia. Clinical findings vary according to the extent and severity of the disease. Recently, Lee et al. proposed a disease severity-based classification for DC, dividing it in three different clinical-pathological stages: stage I and II being non-scarring, and stage III representing cicatricial alopecia, without contemplating their trichoscopic findings.^2^ Trichoscopy has shown to be useful in the diagnosis, prognostic evaluation, and treatment monitoring of scalp disorders.^3^ Regarding DC, Verzi et al. reinforced that trichoscopy allows magnification of structures barely visible to the naked eye, clarifying clinical examination uncertainties, and therefore could be a valuable tool for both diagnosis and treatment choice in this still poorly elucidated disorder.^4^ The present article illustrates trichoscopic features of DC, correlating their images to the clinical stages of disease progression.

In earlier stages of the disease, the trichoscopic picture of DC may simulate that of alopecia areata. The presence of follicular and perifollicular lymphocytic infiltrates on the lower parts of terminal follicles^5^, ^6^ explains the trichoscopic resemblance to alopecia areata, a condition in which the intense peribulbar inflammatory infiltrate is often referred to as a swarm of bees. Involvement of the lower portions of the follicle may lead to telogen and consequent hair loss. The follicle is unable to start a new anagen phase and remains empty, accumulating sebum and keratin, thus justifying yellow dots in trichoscopy. Alternatively, inflammation may impair adequate hair shaft formation.^6^ Weakened shafts break, leading to the formation of broken hairs and black dots. Recently, exclamation mark hairs,^7^ a trichoscopic feature typically associated to alopecia areata, have been described in early DC (Fig. 1). Importantly, such features indicate that this stage is still non-scarring and that hair regrowth is possible with adequate treatment. This non-scarring aspect is represented by Lee et al. in clinical stages I and II.^2^ Nevertheless, it is pointed in the conclusion of their article that DC underdiagnosis is possible, especially at early stages. Thus, the inclusion of trichoscopy in the criteria would be of great value for better diagnostic accuracy.

**Figure 1:**
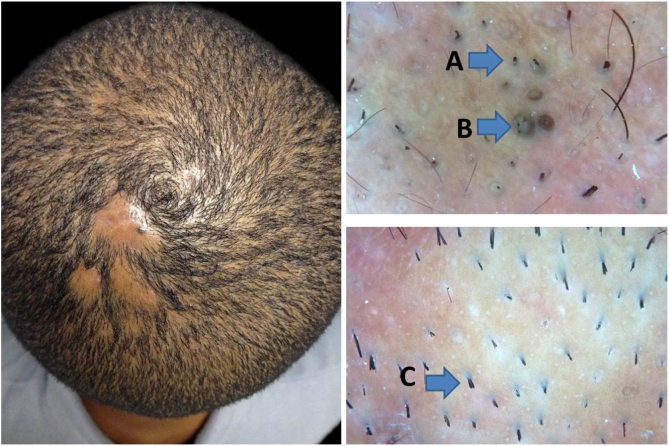
Early stage of dissecting cellulitis, with trichoscopy findings on alopecia areata. A, black dots; B, yellow dots; C, broken hair.

Non-treated DC progresses to the abscedens stage, which presents with severe inflammation and is characterized by the presence of pustules, nodules, and abscesses. In this phase, it is possible to observe three-dimensional yellow dots, which may or may not be imposed over dystrophic hairs, as well as yellow structureless areas.^8^, ^9^ These yellow dots are larger than those described in nonscarring alopecia and in the earlier stage of DC. They have also been described as having a “soap bubble”-like appearance.^8^, ^9^ The yellow structureless areas are “lakes of pus” easily found around hair follicles and are typical of DC (Fig. 2). Pinpoint-like vessels with a whitish halo can also be observed, even though they are not uncommon in other scalp diseases.^7^ In the authors’ experience, if patients are properly treated at this stage, they might recover much of their hair. However, progression to scarring seems inevitable in some areas.

**Figure 2:**
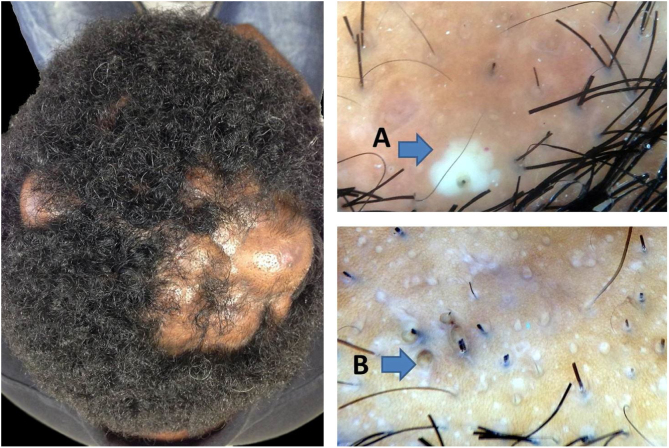
Abscedens stage of dissecting cellulitis. A, yellow structureless area; B, three-dimensional yellow dots.

With the progression of the disease to the fibrotic stage, it is possible to identify extensive dermal fibrosis and destruction of sebaceous glands in histopathology. The fibrotic stage has trichoscopic features that are similar to the end phases of others scarring alopecias, like white areas lacking follicular openings that represent tissue fibrosis, clinically described as shiny patches of alopecia. Another feature that is quite characteristic of advanced DC is the formation of cutaneous clefts. Hair shafts may emerge from such clefts organized into hair tufts with different sizes (Fig. 3).

**Figure 3:**
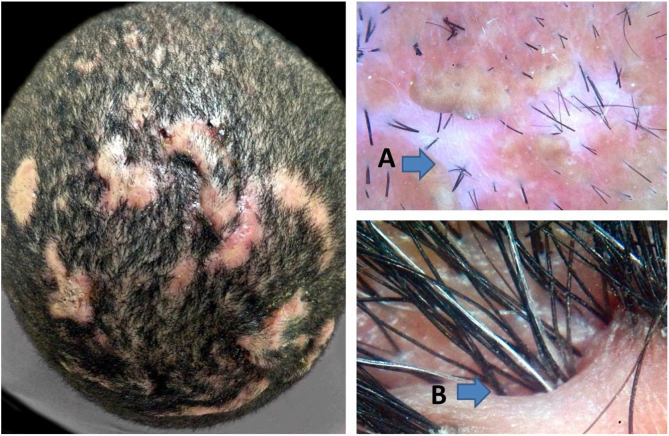
Fibrotic stage of dissecting cellulitis. A, lack of follicular openings; B, cutaneous clefts with emerging hairs.

In conclusion, this article highlights the trichoscopic features of DC focusing on demonstrating their evolutive stages, which could be associated with the clinical criteria proposed by Lee et al.^2^ and enhance trichoscopy's role in diagnosis, treatment choice, and follow-up. This is a suggestion of an additional didactic classification based on the authors’ experience, and some overlap of stages is possible.^4^ The authors emphasize that further investigations are needed in order to confirm the observations. Nonetheless, it is believed that adoption of trichoscopy by dermatologists will refine care of patients with DC, particularly for monitoring patients while on treatment.

## Financial support

None declared.

## Authors’ contributions

Daniel Fernandes Melo: Approval of final version on the manuscript; conception and planning of study; drafting and editing of manuscript.

Luciana Rodino Lemes: Drafting and editing of manuscript.

Bruna Duque-Estrada: Drafting and editing of manuscript.

Rodrigo Pirmez: Drafting and editing of manuscript.

## Conflicts of interest

None declared.
